# Prevalence and factors associated with periodontal disease in patients with diabetes mellitus attending Kiruddu National Referral Hospital, Uganda

**DOI:** 10.11604/pamj.2022.43.202.35971

**Published:** 2022-12-23

**Authors:** Haruna Muhmood Kiryowa, Ian Guyton Munabi, William Buwembo, Charles Mugisha Rwenyonyi, Mark Kaddumukasa, Erisa Mwaka Sabakaki

**Affiliations:** 1 School of Biomedical Sciences, College of Health Sciences, Makerere University, Kampala, Uganda,; 2 School of Health Sciences, College of Health Sciences, Makerere University, Kampala, Uganda,; 3 School of Medicine, College of Health Sciences, Makerere University, Kampala, Uganda

**Keywords:** Diabetes mellitus, glycemic control, HbA1c, periodontal disease

## Abstract

**Introduction:**

patients with diabetes mellitus present with high rates of periodontal disease. Severity and extent of periodontal disease may be directly associated with poor glycemic control. The burden of periodontal disease in patients with diabetes mellitus in Uganda is not documented. This study set out to determine the prevalence and factors associated with periodontal disease in patients with diabetes mellitus attending a national referral hospital in Uganda.

**Methods:**

this was a cross-sectional study involving 264 patients with diabetes mellitus. Data were collected using a pretested questionnaire to assess factors associated with periodontal disease. This was followed by an oral examination to determine the community periodontal index (CPI) and clinical attachment loss (CAL). Laboratory tests included glycated hemoglobin and fasting blood sugar. Factors associated with periodontal diseases were evaluated using logistic regression analysis.

**Results:**

of the 264 participants, 68.9% were females. The average age was 48.9 (SD = 11.0) years. Majority of the participants (32.6%) had diabetes mellitus for 2 to 5 years with oral hypoglycemic drugs being the most commonly (55.7%) used medication. The overall prevalence of periodontal disease was 85%. Univariate analysis revealed that prevalence of periodontal disease was associated with male sex, lower level of education, smoking, oral hygiene practices, poor glycemic control and combined diabetic medication. However, based on multivariate model, this prevalence was only significantly associated with lower level of education: aOR: 10.77 95% CI 1.04-226.38, p=0.05.

**Conclusion:**

periodontal disease is highly prevalent in patients with diabetes mellitus in Uganda, especially those with a lower level of education. All diabetic patients should be screened and managed for periodontal disease. Oral health interventions should also be packaged and presented in a simple language to allow easy comprehension by even the less educated population.

## Introduction

The rate at which the prevalence of diabetes mellitus is increasing has become a concern to mankind [1]. Available data show that about 6.8% of the population worldwide is affected by the disease [2]. A systematic review, Mukamusoni *et al*. [3] reported an average prevalence of 13.7% in the African population. In Uganda, approximately 10.3% of the population have clinically diagnosed diabetes mellitus [4]. Periodontal disease has been identified as one of the early signs of diabetes mellitus [5]. About 95% of patients with diabetes mellitus have some degree of periodontal destruction [6]. Asthana *et al*. [7] revealed that periodontal disease is more prevalent and severe in patients with diabetes mellitus than their counterparts. Periodontal disease may initiate insulin resistance thereby worsening glycemic control [7], which in turn, may lead to an increased extent and severity of periodontal disease [8]. Despite the fact that about 84% of patients with diabetes mellitus in Uganda have poor glycemic control [9], the association between periodontal disease and diabetes mellitus has not been investigated. The present study was aimed at determining the prevalence and factors associated with periodontal disease in patients with diabetes mellitus attending Kiruddu National Referral Hospital in Uganda.

## Methods

**Study design and setting:** this was a cross-sectional study conducted between December 2020 and February 2022 in the Outpatient Department of Kiruddu National Referral Hospital. The hospital is a public facility located in Kampala, capital city of Uganda. The diabetic clinic is one of the 14 specialized out-patients clinics in Kiruddu Hospital. The hospital medical records indicate that the diabetic clinic attends to about twenty new patients on daily basis.

**Study population:** study participants comprised of adult patients with a confirmed diagnosis of diabetes mellitus who provided written informed consent before participating in the study. Individuals who were pregnant, or had a history of periodontal treatment in the last 6 months were excluded from the study. The sample size was estimated using Kish Leslie formula where p = 0.437, e = 0.05 and Z value = 1.645 with statistical power of 90%. The study participants (n=267) were selected using simple random sampling.

**Data collection:** data were collected using a pre tested questionnaire administered by a trained research assistant. The weight in kilograms and height in meters of the participants were determined using a weighing scale (KINLEE, China) and a measuring tape (BYLOO, China), respectively. The measurements were used to calculate the Body Mass Index (BMI). An automated glucometer (Contour Plus, Switzerland) was used to determine the fasting blood sugar while HbA1c levels were determined using an automated analyser (LabonaCheck™ A1cHbA1c, CERAGEM MEDISYS INC, South Korea). An oral examination was carried out by a trained dental surgeon using a mouth mirror and a calibrated periodontal probe (Koushen, China) to determine the Community Periodontal Index (CPI) and the Clinical Attachment Loss (CAL). The data were recorded on the World Health Organization Oral Health Assessment form for adults. The CPI values were recorded as 0, normal; 1 and 2, gingivitis; 3, pockets < 4 mm (mild periodontitis) and 4, pockets > 4 mm (severe periodontitis).

**Definitions:** the outcome variable was periodontal disease. Periodontal disease was defined as presence of either gingivitis or periodontitis. The independent variables included age, sex, level of education, oral hygiene practices, history of active smoking, glycemic control and type of diabetic medication. Level of education was classified into no formal education, primary, secondary and tertiary. Oral hygiene practices were categorized into inadequate (brushing teeth less than twice a day) and adequate (brushing teeth more than twice a day).

**Statistical analysis:** data were entered in Microsoft Excel, cleaned and exported to R (version 4.10) for analysis. Participants were divided into two groups, according to the presence or absence of periodontal disease. Continuous variables were presented as means and standard deviations while categorical variables were presented as percentages. Univariate analysis was used to determine the association between periodontal disease and the independent variables. All independent variables were employed in the multivariate regression analysis because of their potential role in pathogenesis of periodontal disease. P values < 0.05 were considered as statistically significant.

**Ethical considerations:** the study protocol was approved by Makerere University School of Biomedical Sciences Research Ethics Committee (Ref. no. SBS-899) and Uganda National Council of Science and Technology (Ref. no. HS1853ES). The participants gave written informed consent before their enrolment in the study in accordance with Helsinki Declaration [10]. All data were assigned special codes and stored in a password-protected computer. No identifying information of the participants was included in data collection.

**Source of funding:** this study was sponsored by the Government of Uganda through the Makerere University Research and Innovation Fund (MAK-RIF 1).

## Results

**Figure 1:**
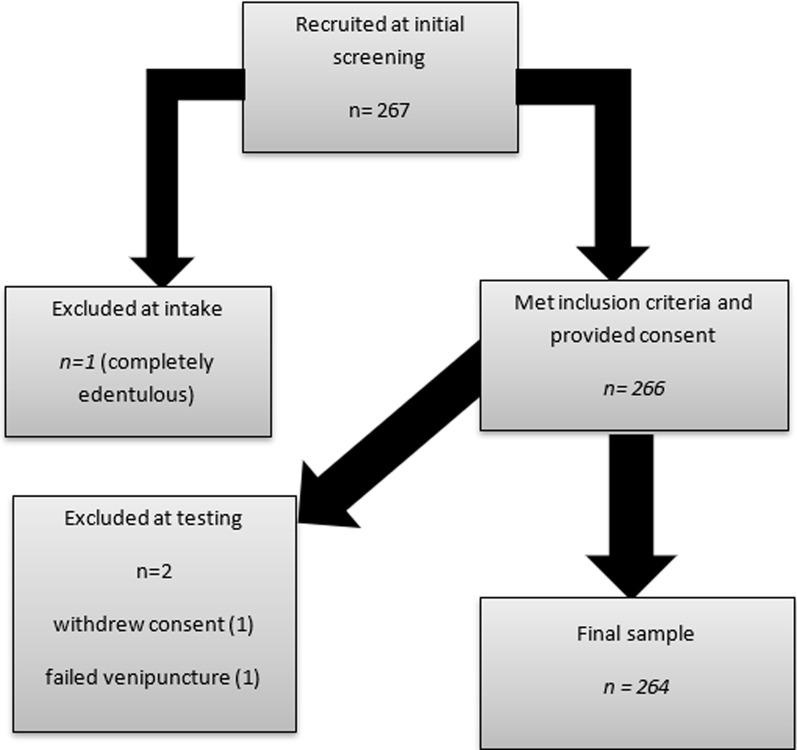
flow chart showing participant recruitment

**General characteristics of the study population:** of the 267 participants, 3 were excluded from the study, for reasons of being completely edentulous (n=1), failing venipuncture (n=1) and withdrawing informed consent (n=1), leaving 264 for the analysis (Figure 1). Most participants (68.9%) were females (Table 1). The average age was 48.9 (SD = 11.0) years. Most participants (78.0%) had attained either primary or secondary education and 14.0% had no formal education. Majority of the participants (93.6%) reported brushing their teeth once (40.6%) or twice (52.6%) a day (Table 1). Only 3.8% reported active smoking. About 97.3% of the participants were earning less than one million Uganda shillings per month. About half (51.6%) of the participants had lived with diabetes mellitus for at least 6 years. The most (55.7%) common form of diabetic medication was oral hypoglycemics while a combination of oral hypoglycemics and injectable insulin was used by 31.4% of the participants. The average body mass index (BMI) was 29.5 (SD=5.78) and mean HbA1c was 8.84 (SD=2.57) (Table 1).

**Table 1:** descriptive characteristics of study participants

Variable	Overall (N=264)
**Sex**	
Male	82 (31.1%)
Female	182 (68.9%)
**Age**	
Mean (SD)	48.9 (11.0)
**Level of education**	
No formal education	37 (14.0%)
Primary	123 (46.6%)
Secondary	83 (31.4%)
Tertiary	21 (8.0%)
**Frequency of teeth cleaning**	
None	4 (1.5%)
Once	108 (40.9%)
Twice	139 (52.7%)
Thrice	13 (4.9%)
**Mode of teeth cleaning**	
None	4 (1.5%)
Brushing stick	4 (1.5%)
Tooth brush	9 (3.4%)
Tooth brush plus tooth paste	245 (92.8%)
Tooth brush, tooth paste and floss	2 (0.8%)
**Income status**	
Low (less than one million Ug. Shs p.m)	257 (97.3%)
High (greater than one million Ug. Shs p.m)	7 (2.7%)
**History of smoking**	
Yes	10 (3.8%)
No	254 (96.2%)
**Mode of smoking**	
Cigarettes	6 (2.3%)
Pipes	2 (0.8%)
Tobacco	2 (0.8%)
None	254 (96.2%)
**Total years with diabetes**	
Less than 1 year	42 (15.9%)
2-5 years	86 (32.6%)
6-10 years	62 (23.5%)
Greater than 10 years	74 (28.0%)
**BMI (kg/m^2^)**	
Mean (SD)	29.5 (5.78)
**Fasting blood sugar**	
Mean (SD)	11.4 (5.53)
**HbA1c (%)**	
Mean (SD)	8.84 (2.57)
**Periodontal status**	
Normal	39 (14.8%)
Gingivitis	66 (25.0%)
Mild periodontitis	84 (31.8%)
Severe periodontitis	75 (28.4%)

Ug: Uganda; Shs: shillings; p.m: per month; BMI: body mass index

**Prevalence of periodontal disease:** most participants (85.2%) had periodontal disease, which was categorized as gingivitis (25%), mild periodontitis (31.8%) and severe periodontitis (28.4%) (Table 1).

**Factors associated with periodontal disease:** based on univariate analysis prevalence of periodontal disease was associated with male sex, level of education, oral hygiene practices, poor glycemic control and combined diabetic medication. From multivariate regression, this association was only significant for lower level of education (aOR: 10.77 95% CI 1.04-226.38, p=0.05; Table 2).

**Table 2:** results of logistic regression analysis of factors associated with periodontal disease in study participants

Dependent: periodontal disease	Attributes	Periodontal disease	Normal	OR (univariable)	aOR (multivariable)
Sex	Male	72 (87.8)	10 (12.2)	-	-
	Female	153 (84.1)	29 (15.9)	1.36 (0.65-3.08, p=0.43)	1.68 (0.72-4.26, p=0.25)
Age	Mean (SD)	49.6 (10.5)	44.4 (12.4)	0.96 (0.92-0.99, p<0.01)	0.96 (0.93-1.00, p=0.03)
Level of education	No formal education	36 (97.3)	1 (2.7)	-	-
	Primary	106 (86.2)	17 (13.8)	5.77 (1.12-105.86, p=0.09)	5.99 (1.13-110.90, p=0.09)
	Secondary	67 (80.7)	16 (19.3)	8.60 (1.65-158.29, p=0.04)	7.54 (1.36-142.02, p=0.06)
	Tertiary	16 (76.2)	5 (23.8)	11.25 (1.64-225.01, p=0.03)	10.77 (1.40-226.38, p=0.04)
Smoking	Yes	9 (90.0)	1 (10.0)	-	-
	No	216 (85.0)	38 (15.0)	1.58 (0.29-29.62, p=0.67)	1.03 (0.15-20.68, p=0.98)
HbA1c	Mean (SD)	8.9 (2.6)	8.4 (2.6)	0.93 (0.80-1.06, p=0.30)	0.89 (0.76-1.03, p=0.12)
BMI	Mean (SD)	29.4 (5.9)	30.2 (5.2)	1.02 (0.96-1.08, p=0.47)	1.01 (0.94-1.07, p=0.81)
Oral hygiene	Inadequate	97 (86.6)	15 (13.4)	-	-
	Adequate	128 (84.2)	24 (15.8)	1.21 (0.61-2.48, p=0.59)	1.04 (0.50-2.22, p=0.92)
Diabetic medication	Both	73 (88.0)	10 (12.0)	-	-
	Insulin	26 (74.3)	9 (25.7)	2.53 (0.91-6.97, p=0.07)	2.57 (0.86-7.64, p=0.09)
	Oral	126 (86.3)	20 (13.7)	1.16 (0.52-2.71, p=0.72)	1.13 (0.49-2.73, p=0.78)

BMI: body mass index

## Discussion

This study set out to investigate the prevalence and factors associated with periodontal disease in adult patients with diabetes mellitus. There was a high prevalence of periodontal disease, which was significantly associated with lower level of education. The prevalence of periodontal disease in this study was 85.2%, with 60.2% of participants having periodontitis and 25%, gingivitis. This prevalence is higher than the 67.8% that was reported in a recent metanalysis involving 3092 patients with diabetes mellitus and 23,494 controls [11]. It was similarly higher than the 45.9% that was reported by Nand *et al*. [12] in a rural Indian population. This difference in prevalence could be attributed to the poor glycemic control in the majority of our participants. Poor glycemic control is one of the factors that has been associated with a high prevalence of periodontal disease in patients with diabetes mellitus [13]. This is true for our study where the mean HbA1c in both groups was greater than 7.0 indicating that most of the participants had poor glycemic control. Inadequate oral hygiene, especially in patients with diabetes mellitus may also have contributed to the increase prevalence of periodontal disease in our study population [14]. Our findings indicate that participants who practiced inadequate oral hygiene had a four percent chance of having periodontal disease compared to those who practiced adequate oral hygiene. Kabali *et al*. also reported a low prevalence of periodontal disease in participants who practiced good oral hygiene [15]. Poor oral hygiene practices accelerate accumulation of plaque, an important reservoir for the periodonto-pathogenic microorganisms.

Males in this study had a sixty-eight percent higher chance of having periodontal disease than females. This is consistent with reports from several other studies that have highlighted the role of male sex as a risk factor for periodontal disease [16,17]. Males usually exhibit relatively poor oral hygiene practices when compared to females [18]. Male participants with poor oral hygiene are more prone to development of periodontal disease than their female counterparts [19]. Older age and smoking have also been identified as risk factors for periodontal disease in male sex [17]. Smoking whether in males or females alters oral microbiome as well causes direct tissue destruction [20]. Though our findings indicate that the chances of having periodontal disease were three percent more in active smokers than in the non-smokers, the number of participants who reported active smoking was small. In addition, the gender differences in smoking status were not evaluated. It is thus difficult to conclude that smoking was an independent risk factor for development of periodontal disease in the male sex.

This study investigated the association between age and periodontal disease. We note that for every unit decrease in age, there was a 4% decrease in odds of having periodontal disease. Eriksson *et al*. have reported ageing to be associated with an increased incidence of periodontal disease [21]. On the other hand, Pranckeviciene *et al*. reported that periodontal disease was more prevalent in the younger patients with diabetes mellitus [22]. However, that particular study was conducted in participants with type one diabetes mellitus whose early exposure to chronic glycaemia predisposed them to higher risks of developing periodontitis [23]. Much as our findings suggest that participants with periodontal disease were slightly older than those without periodontal disease, there was no significant variations in the mean age of the two populations. Periodontal disease has been reported to be more common in the older individuals. Though the risk factors for periodontal disease in the elderly are no different from the younger population, it is important that older people are keeping their teeth longer hence prolonging their exposure to periodontopathogenic bacteria. In addition, older individuals may present with a number of systemic conditions which have a direct or indirect link to the pathophysiology of periodontal disease.

In this study, participants who had no formal education were six times more likely to have periodontal disease than those who had attained primary education. The odds for a person with no formal education having periodontal disease increased for every additional increase in level of education. This was statistically significant (p value = 0.04). Our findings are consistent with Masriadi *et al*. who reported low level of education to be a risk factor for periodontal disease [24]. The level of education and income status are one of the measures of socioeconomic status. Individuals with low level of education are more likely to be poor and unable to afford periodontal treatments which are in most cases expensive, thereby increasing chances of having periodontal disease [25]. Socioeconomic factors like primary education and low social class have been reported to be associated with a greater prevalence of periodontal disease in the adult population [26]. This is true for our study where 97.3% of the participants were earning less than one million Uganda shillings per month which is less than 350 US Dollars per month (Table 1).

Participants using combined therapy of oral hypoglycemics and insulin in this study were twice more likely to have periodontal disease than those who were using insulin alone, and the chances were thirteen percent when compared to those who were using oral hypoglycemics. We opine that this may be related to the association between type of anti-diabetic medication and glycemic control [27,28].

This study had some limitations. It employed a cross-sectional study design to determine the factors associated with periodontal disease. A case-control design would have been the ideal design for this study. The study was also conducted during the COVID-19 epidemic. This might have affected the results of this study.

## Conclusion

Periodontal disease is highly prevalent in patients with diabetes mellitus in Uganda, especially those with a lower level of education. All diabetic patients should be screened and managed for periodontal disease. Oral health interventions should also be packaged and presented in a simple form to allow easy comprehension by the target population.

## What is known about this topic

- There is a high prevalence of periodontal disease in patients with diabetes mellitus;
- The factors associated with periodontal disease in patients without diabetes mellitus increase the risk for development of this condition in patients with diabetes mellitus.

### What this study adds

- Unlike the general consensus that diabetes mellitus mainly affects individuals of the medium to high socioeconomic status, most of the participants from this study were of low socioeconomic status; this is evidenced by the lower level of education and low-income status;
- Apart from the common factors associated with periodontal disease in both patients with and without diabetes mellitus, type of diabetic medication may play an important role in predisposing patients with diabetes mellitus to increased risk of development of periodontal disease; further studies need to be conducted to confirm this association.
